# Assessing the Effectiveness of a Community Intervention for Monkeypox Prevention in the Congo Basin

**DOI:** 10.1371/journal.pntd.0001356

**Published:** 2011-10-18

**Authors:** Amira A. Roess, Benjamin P. Monroe, Eric A. Kinzoni, Seamus Gallagher, Saturnin R. Ibata, Nkenda Badinga, Trolienne M. Molouania, Fredy S. Mabola, Jean V. Mombouli, Darin S. Carroll, Adam MacNeil, Noelle A. Benzekri, Cynthia Moses, Inger K. Damon, Mary G. Reynolds

**Affiliations:** 1 Epidemic Intelligence Service, Centers for Disease Control and Prevention, Atlanta, Georgia, United States of America; 2 Poxvirus and Rabies Branch, Centers for Disease Control and Prevention, Atlanta, Georgia, United States of America; 3 International Conservation and Education Fund, Washington, D.C., United States of America; 4 Medicins D'Afrique, Brazzaville, Republic of the Congo; 5 Laboratoire National de Santé Publique, Brazzaville, Republic of the Congo; Federal University of Technology, Nigeria

## Abstract

**Background:**

In areas where health resources are limited, community participation in the recognition and reporting of disease hazards is critical for the identification of outbreaks. This is particularly true for zoonotic diseases such as monkeypox that principally affect people living in remote areas with few health services. Here we report the findings of an evaluation measuring the effectiveness of a film-based community outreach program designed to improve the understanding of monkeypox symptoms, transmission and prevention, by residents of the Republic of the Congo (ROC) who are at risk for disease acquisition.

**Methodology/Principal Findings:**

During 90 days, monkeypox outreach was conducted for ∼23,860 people in northern ROC. Two hundred seventy-one attendees (selected via a structured sample) were interviewed before and after participating in a small-group outreach session. The proportion of interviewees demonstrating monkeypox-specific knowledge before and after was compared. Significant gains were measured in areas of disease recognition, transmission, and mitigation of risk. The ability to recognize at least one disease symptom and a willingness to take a family member with monkeypox to the hospital increased from 49 and 45% to 95 and 87%, respectively (p<0.001, both). Willingness to deter behaviors associated with zoonotic risk, such as eating the carcass of a primate found dead in the forest, remained fundamentally unchanged however, suggesting additional messaging may be needed.

**Conclusions/Significance:**

These results suggest that our current program of film-based educational activities is effective in improving disease-specific knowledge and may encourage individuals to seek out the advice of health workers when monkeypox is suspected.

## Author Summary

Human monkeypox is a potentially severe illness that begins with a high fever soon followed by the development of a smallpox-like rash. Both monkeypox and smallpox are caused by infection with viruses in the genus *Orthopoxvirus*. But smallpox, which only affected humans, has been eradicated, whereas monkeypox continues to occur when humans come into contact with infected animals. There are currently no drugs specifically available for the treatment of monkeypox, and the use of vaccines for prevention is limited due to safety concerns. Therefore, monkeypox prevention depends on diminishing human contact with infected animals and preventing person-to-person spread of the virus. The authors describe a film-based method for community outreach intended to increase monkeypox knowledge among residents of communities in the Republic of the Congo. Outreach was performed to ∼23,600 rural Congolese. The effectiveness of the outreach was evaluated using a sample of individuals who attended small-group sessions. The authors found that among the participants, the ability to recognize monkeypox symptoms and the willingness to take ill family members to the hospital was significantly increased after seeing the films. In contrast, the willingness to deter some high-risk behaviors, such as eating animal carcasses found in the forest, remained fundamentally unchanged.

## Introduction

Human monkeypox (MPX) is caused by infection with *Monkeypox virus* (MPXV), a member of the *Orthopoxvirus* genus in the family *Poxviridae*. The clinical manifestations of severe MPX bear pronounced similarity to those of smallpox, though fatality rates are consistently lower for monkeypox [1]–[4]. Smallpox has now been eradicated but MPX, a zoonosis, continues to be an endemic disease threat in large areas of the Congo Basin, principally in forested regions of the Democratic Republic of the Congo. It also occurs sporadically in other parts of West and Central Africa [1], [5], [6]. A study published in 2010 reported the cumulative incidence of monkeypox in a disease endemic region of the Democratic Republic of the Congo (DRC) as 5.5 per 10,000—substantially higher than 2 decades prior—with children (under 15 years of age) constituting the greatest proportion of the population affected by this disease [6], [7].

Contact with wildlife, which is routine throughout most of the Congo Basin, is believed to increase the risk for human infection with monkeypox virus [8]. The virus is also communicable between people. During the era of eradication, smallpox vaccine was shown to provide protection against monkeypox infection, but smallpox vaccinations ceased in Africa in 1980. Conventional, second-generation smallpox vaccines (such as ACAM2000™, the vaccine currently licensed for use in the United States) are not currently recommended for widespread use in the region, in part, due to safety concerns stemming from high rates of HIV prevalence. As smallpox vaccine-derived immunity wanes across communities throughout the Congo Basin, monkeypox disease incidence is anticipated to increase.

Blindness due to corneal scarring (typically unilateral), and superficial skin discolorations and scaring have been reported as sequelae of infection for monkeypox [9], [10]. Severe complications of illness include secondary bacterial infections of the skin, bronchopneumonia, dehydration, and encephalitis. Complications appear to occur more frequently in children and in individuals who have not had prior vaccination against smallpox [3], [11]. There are no pharmaceutical therapies approved to treat MPX, and use of conventional licensed vaccines (such as ACAM 2000™) is limited due to safety concerns. Monkeypox remains a disease of persons in impoverished rural areas, and disease control hinges on deterring zoonotic exposure to the virus and, barring that, interrupting person-to-person spread [8].

The 2010 Integrated Disease Surveillance and Response technical guidelines for Africa—jointly produced by the World Health Organization (WHO) and the US Centers for Disease Control and Prevention (CDC)— stipulate that early identification and reporting infectious disease hazards such as monkeypox should be a core function of community-level health systems [12]. In many resource poor areas, however, community health workers are rare, which shifts the burden of early hazard detection and reporting to the community members themselves. There are many obvious challenges to the engagement of community members for this task, not the least of which is identifying effective and lasting means for imparting basic information about an infectious disease hazard, how to avoid the disease, and why it is important to alert the medical community when occurrence of the disease is suspected.

In 2003, there was an outbreak of monkeypox identified in the town of Impfondo in the Likouala region of the Republic of the Congo (ROC) [10]. A subsequent serosurvey performed in the region revealed seroprevalence levels for *Orthopoxvirus* antibodies ranging from 23–83% in various villages and towns throughout Likouala, suggesting high rates of exposure to *Orthopoxviruses* in a population that, for the most part has not benefited from the smallpox vaccine [13]. These observations highlighted the need for initiation of rash illness surveillance in the area, focusing primarily on the identification of human monkeypox. Along with this surveillance activity, a comprehensive program of community outreach and education was initiated by CDC and partners, with the dual intent of stimulating community participation in the surveillance activity and providing community members with basic knowledge to protect themselves against the disease.

Film-based approaches have previously been shown to be effective in imparting health messages to members of rural communities in Africa [14]–[16]. Beginning in 2008, a series of educational films addressing monkeypox were produced by the International Conservation and Education Fund (INCEF), in conjunction with CDC. Two of these films were produced specifically for the purposes of educational outreach to members of communities at risk for monkeypox in the Likouala region. During the months of June–October in 2009, INCEF educators performed community outreach for monkeypox in 16 towns and villages in Likouala, and conducted a real-time evaluation of the effectiveness of the education program in increasing community member's basic knowledge of the disease and their intended future behaviors relevant to risk reduction, such as diminishing contact with suspected zoonotic hosts of MPXV [17]–[19]. In addition, the basic retention of knowledge about monkeypox was assessed among persons who had participated in a pilot program of outreach performed 6 months to a year prior. We report the results of these evaluations.

## Methods

### Films and Content

Two films addressing monkeypox were produced by INCEF personnel with technical input provided by personnel from the US Centers for Disease Control and Prevention, the Republic of the Congo's Ministry of Health (*Ministere de la Sante et de la Population*), and the WHO office in Brazzaville. The films (“*Monkeypox Testimonies*” and “*Understanding Monkeypox*”) feature recognizable members of the local community (i.e., health professionals and individuals who had been affected by monkeypox). The first film (13 minutes in length) covers topics related to monkeypox recognition, modes of acquisition, and consequences (e.g., costs and sequelae); the second (12 minutes) covers topics related to virus transmission, disease prevention and the importance of seeking medical care. Both films feature individuals speaking in, or dubbed in, Lingala (a local language). Monkeypox educational materials can be viewed at http://www.incef.org/.

### Dissemination Method

A team of 2 facilitators, one an experienced community outreach coordinator and one versed in health education, conducted each outreach session. Two teams performed all the outreach described in this work. The lead facilitator trained the other team members in outreach and interview methods. A typical outreach mission entailed a team's travelling, by foot, bicycle or boat, to towns or outlying communities, performing outreach at a single location for 2–4 nights at a time. The use of portable –rechargeable—batteries as power sources for projection equipment allowed a team to remain in the field for several days at a time, or weeks at a time if a portable generator was also utilized.

Written permission from local government officials (prefect, sous-prefect) was obtained prior to the team's departure into the field, and census data for outreach locations were collected.

**Figure 1:**
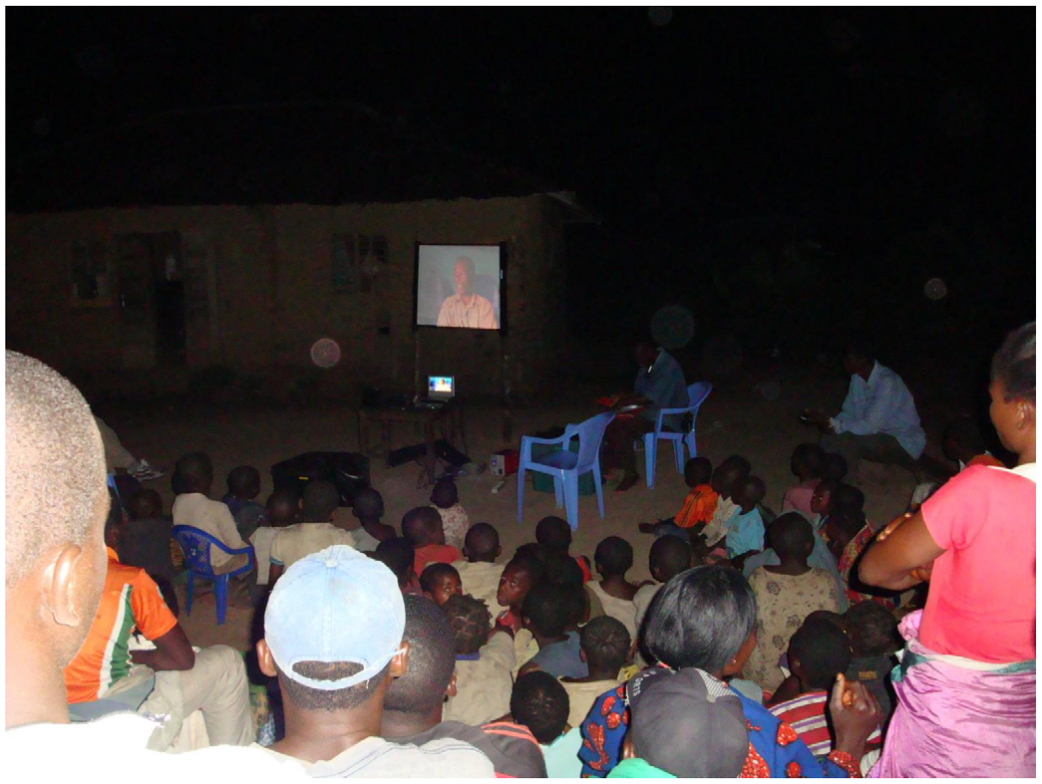
Monkeypox outreach session in the village of Bonzale, ROC, May, 2009. (Photo EAK. Monkeypox educational materials can be viewed at http://www.incef.org/.)

Upon arrival at an outreach location, educators met with village leaders to obtain verbal permission to conduct the outreach. Outreach activities began with small-group sessions involving 10–100 persons per group (depending on the size of the village), with participants separated into the following 5 groups: children <13 years old (these individuals did not participate in the evaluation component of this activity), individuals 13–18 years old, males ≥18–35 years old, males >35, and females ≥18 years old. Small-group education sessions were conducted individually (one at a time) over the course of two days, typically during the day in a suitable building or structure (church, school, etc). Those who volunteered to participate in the small-group sessions were asked to form a queue and members were admitted until the relevant quota was obtained. After completion of the small-group sessions, additional outreach sessions were held at night for the entire village (or neighborhood within larger towns) (**Figure 1**).

Both small and large-group sessions began with a general discussion of monkeypox. Then the first film in the module was shown, followed by a 15–45 minute discussion about the information presented. The second film was then shown, again with subsequent discussion. Facilitators recorded (on paper) anecdotes and comments made by participants during the question and answer period.

### Evaluation Method

Prior to viewing the films, approximately 10% of participants in the small-group discussions (described above) were selected to complete pre- and post-screening interviews. Volunteers for the interviews identified themselves by raising hands, after which the facilitators selected the appropriate number of interviewees based on convenience, quota- selection process. The interview tool consisted of a series of open-ended or yes/no questions addressing material presented in the films (Text S1). Each person was questioned individually out of earshot of others. Questions were read out loud in Linguala (a local language) by one facilitator, and responses were recorded on a paper questionnaire sheet by the second. Questions that were asked prior to screening the film addressed the individual's basic knowledge of monkeypox, including the principal disease symptoms, and modes of virus transmission. As well, questions were asked that pertained to the individual's current (or past) behaviors regarding health care-seeking practices and the handling of primate and rodent carcasses. After viewing the films, individuals were again questioned about their basic knowledge of monkeypox (as above). In addition, interviewees were queried as to their intended future behaviors with respect to when they would seek health care for themselves or a family member, and how they would handle primate and rodent carcasses. Respondents also provided their age, sex, occupation or school attendance status, and current village of residence (if other than the village or town in which the films were shown).

### Ethics Statement

The purpose of the proposed activity was to evaluate an educational film designed to deliver health messages about monkeypox to communities in ROC, in order to determine whether the messages were understood by the audience. A written protocol describing the evaluation was reviewed by Human Subjects Research Advisors at the National Centers for Zoonotic and Vector Borne Diseases at CDC, who determined that the work did not involve research as defined under 45 CFR 46.102(d). Prior to engaging volunteers, the evaluation methods, purpose, and voluntary nature of the evaluation was described by the health educators to prospective participants and verbal consent was obtained.

### Analysis

Two hundred eighty-two questionnaires were generated, of which 271(96.1%) were determined complete for analysis (i.e., interviewees provided an answer for at least one question both prior to and again after viewing). Data analyses were based on affirmative responses. Comparisons of responses pre- and post- screening were calculated with a McNemar's Test to account for matched-pair data. Associations between groups were calculated using Pearson's Chi-Square or Fisher's Exact tests. A p-value of <0.05 was considered statistically significant. All statistical analysis was performed in PASW Statistics 18 software (SPSS, Chicago, IL, USA).

Intervention locations were geo-referenced using maps available from the UNHCR GIS and Mapping Unit in Kinshasa and Google Earth (UNHCR [2010] DRC Refugees in the Republic of Congo. Kinshasa, DRC.) Locations were divided into geographic sectors based on proximity to large towns (Impfondo, Dongou, Enyelle) and ease of travel by road or river.

## Results

### Outreach and Evaluation Population

**Figure 2:**
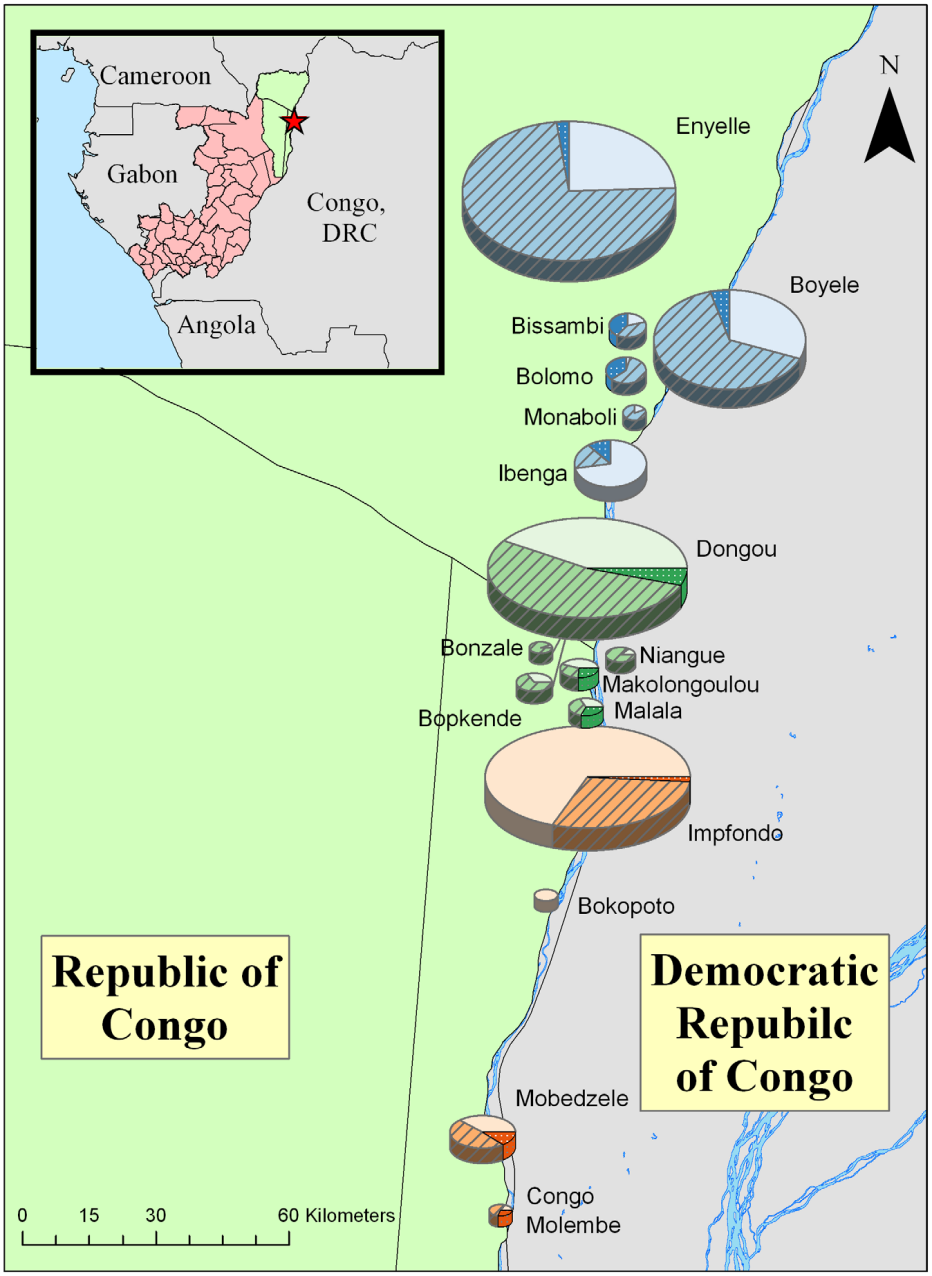
Community participation in the monkeypox outreach film screening events, COG, 2009. Box inset highlights on the area of ROC in which the outbreak was performed. Each circle shown on the expanded map is a proportionally-sized representation of the villages and towns where films were shown. Villages and towns in the north, central and southern sectors are represented in blue, green and orange, respectively. The percent of the population attending the film presentations is represented by stripped and double-hatched sections, with the latter corresponding to small-group participants. Approximately 10% of those in the small groups were interviewed before and after seeing the films.

During a 47 day period between May and June, 2009, educators performed outreach in 7 locations (4 towns and 3 villages) to ∼19,000 people. (In towns— locations with populations >1000 – multiple presentation were made in order to cover individual neighborhoods.) A further ∼4500 persons, from 9 locations (2 towns, 7 villages), received outreach during a 43 day period between July and October of that same year. For both periods approximately half of the enumerated population residing in the area covered attended the outreach activities (47% for the former period and 64% for the latter). The geographic zone of outreach coverage extended from Enyelle in the north to Congomelembe in the south, encompassing 3 sectors within Likouala—north, south and central (**Figure 2**). The northern sector included the greatest number of outreach attendees (11,057), followed by the southern sector (7,860) then the central sector (4,943).

Prior to conducting outreach for the entire village or town, roughly equal numbers of individuals representing the 5 age/sex categories (described above) were selected to participate in structured small-group discussions. Further, within four of these small groups, approximately 1 out of 10 persons was recruited (selected among volunteers until the quota was met) to complete pre and post-screening interviews (**Table 1**). The mean age of persons completing the pre- and post-test questionnaires was 33 years overall. This was consistent across the three geographic sectors. A somewhat higher percentage of males than females completed questionnaires (57%), though the distribution neared equivalence in the central sector.

**Table 1:** Population participation in the 2009 monkeypox outreach program in the Likouala Region of ROC.

		Completed Evaluation Interview					
Location	Attendance at outreach event (%)*	Number	Juveniles†	Adults†	Mean age in years (range)	Male participants (% of total)	Participated in pilot outreach‡
**South**							
Bokopoto	262 (99)	24	4	20	28.0 (13–47)	13 (54)	16
Congomalembe	189 (76)	11	2	9	42.0 (17–64)	7 (64)	0
Impfondo	6311 (32)	7	0	7	29.1 (20–41)	5 (71)	4
Mobedzele	1098 (35)	15	5	10	35.9 (13–70)	9 (60)	0
*Sub-total*	7860 (35)	57	11	46	33.8 (13–70)	34 (60)	20
**Central**							
Bonzale	169 (66)	12	0	12	39.2 (20–41)	8 (67)	10
Bopkende	97 (93)	10	1	9	34.9 (18–50)	4 (40)	2
Dongu	4296 (55)	39	7	25	34.1 (15–65)	18 (47)	12
Makolongoulou	146 (87)	20	5	14	33.3 (14–67)	10 (53)	10
Malala	121 (52)	11	3	7	31.4 (13–53)	6 (54)	5
Niangue	114 (40)	13	6	6	22.4 (13–35)	9 (69)	3
*Sub-total*	4943 (56)	105	22	73	32.5 (13–67)	55 (53)	42
**North**							
Bissambi	187 (66)	16	0	13	36.2 (19–54)	9 (56)	3
Bolomo	312 (93)	17	1	14	34.8 (18–55)	10 (59)	6
Boyele	3269 (69)	36	8	28	28.9 (13–65)	22 (61)	2
Enyelle	7024 (75)	13	2	10	40.7 (29–63)	7 (64)	0
Ibenga	178 (17)	12	2	8	31.4 (17–43)	8 (67)	5
Monaboli	89 (81)	15	2	13	36.9 (13–60)	7 (47)	1
*Sub-total*	11,057 (69)	109	13	86	32.1 (13–65)	65 (60)	17
**TOTAL**	**23,860 (51)**	**271** §	**48**	**205**	**32.9 (13–70)**	**154 (57)**	**79**

*Percentage represents proportion of the town or village that attended the outreach. Population figures are approximate, based on 2007 census information.

**†:** Juveniles here are 13–18 years old; adults are ≥18 years old.

**‡:** Includes male and female interviewees.

**§:** Age was not recorded for 18 interviewees.

In each of the three sectors monkeypox ‘experienced’ interviewees were identified. These were individuals who at the time of the 2009 outreach professed a prior knowledge of monkeypox that had been obtained during the prior 12 months from a health professional or education specialist. Whether the interviewee recalled INCEF by name or not, all educational encounters were presumed to be INCEF encounters as no other outreach for monkeypox was being undertaken in the region. The northern sector had the lowest proportion of ‘experienced’ interviewees which accorded with our expectations based on the locations of pilot outreach activities (performed 6 months to one year prior).

### Knowledge Gained

The pre-and post-test questionnaires addressed the interviewee's knowledge of the signs and symptoms of monkeypox (‘disease recognition’), and the principal modes of inter-human and zoonotic virus transmission (‘disease transmission’), as well as his or her past and future (intended) behaviors with regard to found animal carcasses (‘zoonotic risk’) and seeking of health care (‘health seeking’) (**Table 2**). For most knowledge and behavior subject areas— with the exception of issues relating to ‘zoonotic risk’— the proportion of interviewees who exhibited enhanced knowledge or a stated intention toward a constructive behavior after the outreach was statistically significant. For example, the proportion of interviewees who could recognize lesions on the palms of the hands and soles of the feet as a sign of monkeypox increased from 14 to 51% (p<0.001). As well, the proportion who said that they would avoid touching an animal that they found dead in the forest (with no known cause of death) increased from 23 to 61% (p<0.001). A significant gain in the proportion of interviewees who could identify the possibility of monkeypox virus transmission via objects that had been used by a patient (linens, clothing) was observed, but the final proportion of individuals exhibiting the gain remained low (14%). For multiple subject areas, gains in knowledge exceeded 30%, and for 2 (the ability to identify one sign or symptom of monkeypox, and the willingness to take a family member with monkeypox to the hospital), the final proportion of the interviewees who had improved knowledge or who professed a willingness to perform a constructive behavior (such as willingness to take an ill family member to a hospital) exceeded 85%.

**Table 2:** Comparison of interviewee responses to questions before and after attending a monkeypox outreach session.

Question subject area	Answered “yes” before viewing films*number (%)	Answered “yes” after viewing films*number (%)	*p-value* †
**Disease recognition**			
Q1. Knows at least one symptom of MPX	133 (49)	258 (95)	<0.001
Q2. Can name rash and fever are symptoms of MPX	29 (11)	86 (32)	<0.001
Q3. Knows MPX lesions can occur on palms and soles	39 (14)	137 (51)	<0.001
**Disease transmission**			
Q4. Can occur by direct contact with an ill person	77 (28)	156 (58)	<0.001
Q5. Can occur by contact with soiled items (fomites)	6 (2)	38 (14)	<0.001
Q6. Can occur by contact with an ill animal	65 (24)	172 (64)	<0.001
Q7. Can occur by contact with an dead animal	20 (7)	50 (19)	<0.001
**Zoonotic risk** ‡			
Q8. Has eaten/would eat primate carcass found in forest	29 (11)	30 (11)	1.000
Q9. Has sold/would sell primate carcass found in forest	11 (4)	11 (4)	1.000
Q10. Has eaten/would eat rodent or squirrel carcass found in forest	88 (33)	43 (16)	<0.001
Q11. Has sold/would sell rodent or squirrel carcass found in forest	18 (7)	9 (3)	<0.001
**Risk mitigation**			
Q12. Would take family member with MPX to hospital	130 (48)	236 (87)	<0.001
Q13. Would take family member with MPX to traditional healer	4 (2)	3 (1)	1.000
Q14. Would avoid direct contact with an ill person	71 (26)	159 (59)	<0.001
Q15. Would avoid touching soiled items (fomites)	8 (3)	35 (13)	<0.001
Q16. Would avoid touching animal carcasses found in forest	63 (23)	164 (61)	<0.001

*Interviewee answered ‘yes’ to the question, or had otherwise affirmed knowledge (i.e, was able to select rash, or other symptom as being associated with monkeypox.)

**†:** Comparisons of responses pre- and post- screening were calculated with a McNemar's Test to account for matched-pair data.

**‡:** Questions refer to animal carcasses found in the forest for which there was no obvious cause of death (e.g., trauma). Prior to seeing the films, interviewees were asked whether they had engaged in the behavior at any time in the past. After seeing the film, interviewees were asked what they would do in the future if they were to find an animal carcass in the forest.

However, when queried about past behaviors and intended future behaviors relevant to collection of wild rodents and primates, some intended behaviors remained invariant. For example, while few interviewees (n = 29, 11%) stated that they had in the past eaten a monkey that they had found dead in the forest, the number remained essentially unchanged after interviewees saw the films and were then asked if they would engage in the behavior in the future (n = 30, 11%). This trend was consistent for collection and for sale of found primate carcasses as well. Considerably more interviewees stated that they had eaten rodents or squirrels that they had found dead in the forest (n = 88, 33%), but here after viewing the films the proportion who said that they would do so again after diminished by a significant fraction (n = 43, 16%) (p<0.001). The result was similar with regard to intended sales of rodent or squirrel carcasses.

Of interest, both before and after viewing the films, very few (n = 4, 3, respectively) said that they would take an ill family member (suspected of having monkeypox) to a traditional healer.

### Trends by Age, Sex, and Location

**Figure 3:**
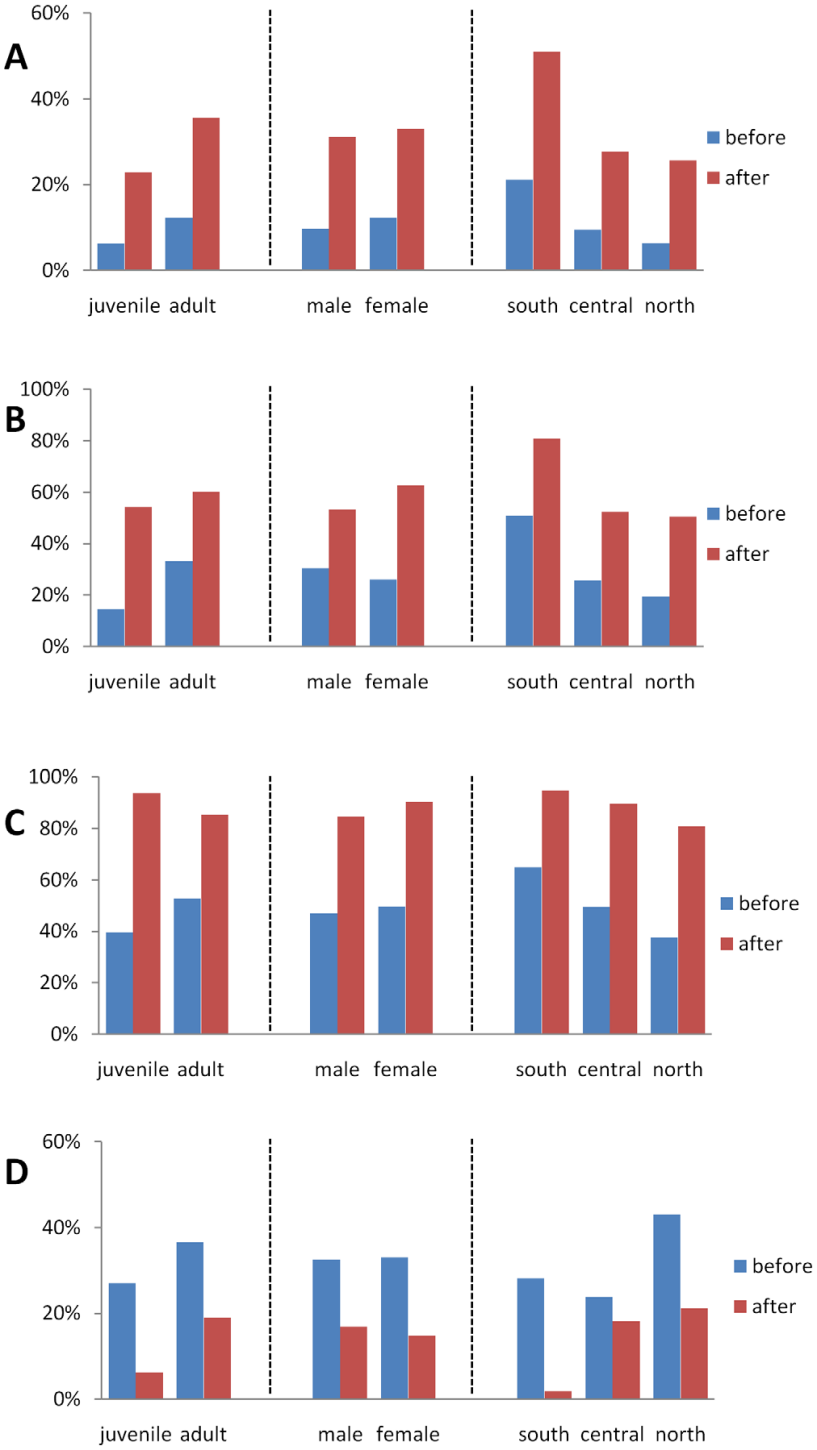
Comparison of interviewees responses to monkeypox-related questions before and after participation in the outreach. Results are stratified by age group, gender, and geographic location. Bars show the percent of respondents within each category who indicated that, (A) monkeypox virus infection involves fever followed by skin rash; (B) monkeypox is transmitted by direct contact; (C) he/she would take a family member suspected of having monkeypox to the hospital, and; (D) he/she has eaten (before) or would eat (after) a rodent or squirrel carcass found in the forest.

When looking at response patterns in light of various categories of interviewees—tabulated by sex, age or geographic location—we found that geographic location was the variable most often significantly associated with the response outcome. (The association was found for 12 questions, asked either before or after film screening.) In general, interviewees in the southern sector were significantly more knowledgeable than those in the central or northern sector about monkeypox symptoms and disease transmission, and this group had the lowest proportion of interviewees indicating prior and intended future behaviors relating to the consumption of found animal carcasses. Beyond this, few categorical differences were noted when responses were evaluated with regard to sex or age. However, after seeing the films, female interviewees were significantly more likely than male counterparts to say that they would avoid direct contact with an ill person (66% vs. 53% p = 0.034). As well, prior to seeing the films juveniles (<15 years) were less aware than adults of the risk of disease transmission from direct contact with an ill person (15% vs. 33%, p = 0.011) and were less likely to avoid contact with a sick person (10% vs. 32%, p = 0.003). However, after seeing the films juveniles reported being significantly less likely than adults to say that they would consume the carcass of a found rat or squirrel (6% vs. 19%, p = 0.032). Responses to 4 exemplar questions, broken down by participant category, are shown in **Figure 3**.

### Retention of Prior Knowledge

One factor which seemed to influence the how interviewees answered questions prior to viewing the films, was whether he or she had had previous experience with monkeypox outreach. Within the pool of interviewees, 79 persons were identified as ‘experienced’, the remaining ‘naïve’ (**Table 3**). Prior to viewing the films, experienced interviewees were significantly more likely to know the symptoms of monkeypox and that the virus is transmissible by direct contact with someone who is ill. Experienced interviewees were also significantly more likely to say prior to seeing the films that monkeypox could be avoided by avoiding direct contact with someone who is ill and by avoiding contact with animals found dead in the forest. For the most part, these differences became less apparent or unapparent after the current intervention took place.

**Table 3:** Comparison of ‘experienced’ and ‘naïve’ interviewee responses before and after attending a monkeypox outreach session.

	Answered “yes” before viewing films*				Answered “yes” after viewing films*			
Question subject area	naïven (%)	experienced n (%)	chi-square	*p-value* †	naïven (%)	experienced n (%)	chi-square	*p-value* †
**Disease recognition** ‡								
Q1.	65 (34)	68 (86)	61.1	<0.001	182 (95)	76 (96)	0.244	0.621
Q2.	10 (5)	19 (24)	20.8	<0.001	52 (27)	34 (43)	6.58	0.010
Q3.	18 (9)	21 27	13.5	<0.001	85 (44)	52 (66)	10.40	0.001
**Disease transmission**								
Q4.	43 (22)	34 (43)	11.7	0.001	114 (59)	42 (53)	0.88	0.347
Q5.	4 (2)	2 (3)	0.5	0.820	22 (12)	16 (20)	3.59	0.058
Q6.	26 (14)	39 (49)	39.4	<0.001	113 (59)	59 (75)	6.05	0.014
Q7.	6 (3)	14 (18)	17.5	<0.001	31 (16)	19 (24)	2.32	0.127
**Zoonotic risk**								
Q8.	5 (3)	24 (30)	2.2	0.135	5 (3)	25 (32)	2.46	0.111
Q9.	9 (5)	2 (3)	0.7	0.414	9 (5)	2 (3)	0.67	0.414
Q10.	74 (39)	14 (18)	2.8	0.097	35 (18)	8 (10)	11.06	0.001
Q11.	15 (8)	3 (4)	1.5	0.228	8 (4)	1 (1)	1.47	0.455
**Risk mitigation**								
Q12.	59 (31)	71 (90)	78.4	<0.001	168 (88)	68 (86)	0.10	0.751
Q13.	2 (1)	2 (3)	0.9	0.583	2 (1)	1 (1)	0.03	1.000
Q14.	42 (22)	29 (37)	6.4	0.012	117 (61)	42 (53)	1.40	0.238
Q15.	5 (3)	3 (4)	0.3	0.695	23 (12)	12 (15)	0.51	0.474
Q16.	21 (11)	42 (53)	55.9	<0.001	103 (54)	61 (77)	13.01	<0.000

*Interviewee answered ‘yes’ to the question, or had otherwise affirmed knowledge (e.g., was able to select rash, or other symptom as being associated with monkeypox.)

**†:** Associations between groups were calculated using Pearsons Chi-Sqare or Fisher's Exact tests. Naïve n = 192; experienced n = 79.

**‡:** Textual description of questions is provided in Table 2.

### Anecdotes

The open-end responses to interview questions offer additional insight into the film participants' knowledge and behavior regarding monkeypox. A summary of pertinent responses is given in **Table 4**. The most common anecdotes recorded regarded the cultural norms associated with hunting, selling, and eating forest animal products (bushmeat). These often involved perceptions and beliefs surrounding the disease including conspiracies involving the introduction of the virus to the area or a disbelief in the existence of disease. Also implicated were the roles of hunters in bringing disease into communities and the necessity to collect forest animal products the only source of protein or income for family members. Additional themes from these responses included a belief that the disease was not present in one's particular village, or it was not necessary to change behaviors until the disease emerged in that area. Confusion also seems to exist between the role of animals from the forest (primates) and villages (rodents) in spreading disease. Some respondents also expressed a desire for vaccination and treatment options in addition to basic education.

**Table 4:** A sample of qualitative anecdotes from open-ended interview responses.

Age	Gender	Sector	Recorded anecdote
adult	Male	Central	“*I am a hunter, I used to eat dead animals found in the forest, but with the onset of this disease I am obliged not to touch them*.”
adult	Female	South	“*How could we know that the monkey sold in the market is suffering from this disease? Simply tell people not to eat monkey meat*.”
Adult	Male	North	“*Monkeypox doesn't exist, it is a conspiracy by the Ministry of Forest Economy to make us stop eating good monkey meat*.”
Adult	Male	Central	“*I can't leave this meat in the forest because I would be leaving my children hungry at home*.”
Juvenile	Male	South	“*We are accustomed to eating all that we find in the forest, so we are required to be careful now about what we eat*.”
Adult	Female	North	“*The risk to our children is enormous, they walk barefoot and they fight with the mice near their food every morning*.”
Adult	Male	Central	“*It is incorrect to think that no traditional treatment can cure this disease, we have formidable leaves in the forest*.”
Juvenile	Female	North	“*You've only come to tell us about this disease, when will you come to vaccinate us*?”

## Discussion

As of 2007, WHO's Global Health Observatory (http://apps.who.int/ghodata/) estimated that the prevalence of community health workers in the Republic of the Congo was <0.5 per 10,000 persons. (For purposes of comparison, Rwanda in 2004 reported 14 community health workers per 10,000.) In many areas of the country the concentration of community health workers is insufficient to sustain effective surveillance for communicable disease threats, suggesting that it may be necessary to mobilize the community members themselves to assist in identifying early instances of disease. However, there are obstacles to garnering effective participation by community members in these activities. The populations most at risk for monkeypox are likely to be hard to access, due to limited infrastructure for both communication and transportation, and they may be harder yet to mobilize because of intrinsic cultural and linguistic barriers. In this paper, we described a method for culturally-appropriate community-based monkeypox outreach that has been demonstrated to be scalable for large numbers of individuals over a broad geographic expanse, and which we demonstrate to be effective in imparting basic disease-specific knowledge to persons with relatively low levels of health literacy.

The monkeypox outreach program described here was performed in 16 towns and villages in northern Republic of the Congo, and involved people many of whom had little formal education and most of whom had little access to health services. Despite this, among small-group participants we were able to demonstrate substantial gains—and high endpoints percentages – in people's ability to recount the major symptoms of monkeypox (95% could recount at least one major symptom after the outreach, and roughly a third identified both rash and fever) and in their professed willingness to seek healthcare when they suspect a family member has the illness (87% said they would do so after the outreach). We would anticipate that in combination these two elements could have a considerable impact on disease reduction through early case identification and diminished opportunities for community-based transmission of monkeypox virus.

However, there are multiple subject areas for which our messages about disease transmission or risk mitigation strategies resonated less successfully. For example, while the proportion of interviewees who could identify contaminated fomites (bedding, clothing) as a vehicle for transmission was significantly increased after seeing the films, the overall endpoint remained relatively low at 14%. As well, after seeming the films a similarly low proportion (13%) identified avoidance of potentially contaminated items as a means to reduce the risk of contracting monkeypox. Also of note, a small proportion of individuals reported after seeing the films that they would continue to eat the carcasses of dead primates found in the forest (carcasses for which no signs of trauma or cause of death was evident). These same individuals reported having done so in the past; their intended behaviors were essentially unchanged after participating in the outreach. While the numbers of individuals who purportedly engage in such behaviors is relatively small, the activities in question could be of measureable importance to the manner in which virus enters into human communities. Small-group participants seemed far more accepting of avoiding contact with (i.e., eating, selling) the found carcasses of rodents or squirrels. Young people (those under 15 years of age) were significantly more apt to say that they would avoid doing such than older people, after having seen the films.

During this outreach period which took place from May–October, 2009, several villages and towns were included which had also participated in pilot outreach activities during the year prior. The pilot program was constructed with the same core of learning objectives and recommendations, and also utilized film and discussions, but the materials employed were modified by INCEF after the pilot to better conform to local needs. The fact that some interviewees had had prior experience afforded us with some insights into the durability of the knowledge imparted using these methods. Interviewees who we identified as having had prior experience with monkeypox outreach were in particular better able to answer questions addressing disease recognition than were those who had not received outreach during the pilot phase. Much of the pilot-phase outreach took place in the southern sector and indeed interviewees from the south in general displayed more knowledge about monkeypox and more often professed an intention to pursue risk-reduction behaviors than did those from the central or northern sectors.

Attempts were made to minimize potential biases introduced to the survey during selection of subjects and questionnaire design, but the possible influence of volunteer bias (a type of selection bias) and attention bias (a measurement bias) toward inflation of positive findings cannot be entirely overlooked. Whether pronounced or subtle, these biases have the impact of reducing our confidence that findings from this evaluation can be directly extrapolated to the small-group participants or to the broader population of Likouala residents who participated in the outreach. We also cannot discount potential error stemming from the inexperience of this population in participating in interview-driven questionnaires. When interpreting interviewee responses pertaining to intended behaviors, it must be remembered that a person's stated intention of a future behavior may have little to no bearing on what that person is actually apt to do. Measuring these behaviors will be an important objective for future evaluation studies.

The goal of this outreach effort was and is to assist people living in communities at risk for the monkeypox virus to participate in preventing the introduction and spread of disease. The results of this evaluation indicate that our current program of film-based educational activities has been effective in improving disease-specific knowledge in both juvenile and adult community members, and may have the effect of encouraging individuals to seek out the advice of health workers when they are confronted with suspicion of monkeypox. Our next steps involve improving the educational messaging to better inform individuals about all sources of virus transmission (including fomite) and to better encourage risk-mitigation strategies for preventing zoonotic infections. The ultimate measure of the impact of this program will be an increase in community-wide detection and reporting of disease alongside enhanced prevention efforts.

## Supporting Information

Text S1 **English translation of survey questions shown in abbreviated form in** **Tables 2** **and** **3** **.**
(DOCX)
