# Supplementary material for: Assessing the Effectiveness of a Community Intervention for Monkeypox Prevention in the Congo Basin
Source: PLoS Negl Trop Dis. 2011 Oct 18;5(10):e1356. doi: 10.1371/journal.pntd.0001356 (PMC3196471; doi:10.1371/journal.pntd.0001356)
Supplement: Text S1 — English translation of survey questions shown in abbreviated form in Tables 2 and 3 . (DOCX) [file pntd.0001356.s001.docx]

**English translation of survey questions. Responses are shown in Tables 2 and 3.**

Q1. What are the symptoms of monkeypox? (select all that apply)

- High fever
- Skin rash
- Fatigue
- I don’t know
- Other (please explain)

Q2. Response derived from above.

Q3. What is the difference between (how can you distinguish) monkeypox from chickenpox? (select all that apply)

- It is difficult to differentiate (can’t tell them apart)
- Monkeypox is characterized by a high fever followed by skin rash*
- Chickenpox is characterized by superficial skin lesions and simultaneous appearance of fever and rash
- Monkeypox is characterized by the presence of well circumscribed (round) lesions on the palms of the hands and soles of the feet.
- I don’t know
- Other (please explain)

Q4-7. What is the mode of transmission of monkeypox? (select all that apply)

- By direct contact with a person who is ill with the disease
- By contact with soiled objects from a sick person
- By contact with an animal that was found dead in the forest (with no injury or other obvious explanation for death)
- By contact with a sick animal
- I don’t know
- Other (please explain)

Q8, 9. Have you ever found a monkey dead in the forest, if yes, what did you do with it/ if in the future you ever find a monkey dead in the forest what will you do with it?

- Ate it/will eat it
- Collected it/ will collect it
- Touched or manipulated it/ will touch or manipulate it
- Left it/ will leave it
- Sold it/will sell it
- Other (please explain)

Q10, 11. Have you ever found a rodent or squirrel dead in the forest, if yes, what did you do with it/ if in the future you ever find a rodent or squirrel dead in the forest what will you do with it?

- Ate it/will eat it
- Collected it/ will collect it
- Touched or manipulated it/ will touch or manipulate it
- Left it/ will leave it
- Sold it/will sell it

Q12, 13. What do you do when a member of your family contracts this disease/ what will you do in the future if a member of your family contracts this disease? (select all that apply)

- Take him/her to the hospital/clinic
- Take him/her to a traditional healer
- Isolate him/her at home
- Apply traditional medicines
- Other (please explain)

Q14-16. How do you avoid contracting monkeypox? (select all that apply)

- By avoiding direct contact with sick people and sick animals
- By avoiding touching soiled objects
- By avoiding touching, manipulating, or eating animals found dead in the forest
- By avoiding touching, manipulating, or sick animals (with skin lesions)
- I don’t know
- Other (please explain)
